# Supplementary material for: Myc and Tor drive growth and cell competition in the regeneration blastema of Drosophila wing imaginal discs
Source: Development. 2025 Dec 15;152(24):dev204760. doi: 10.1242/dev.204760 (PMC12752512; doi:10.1242/dev.204760)
Supplement: Supplementary information [file develop-152-204760-s1.pdf]

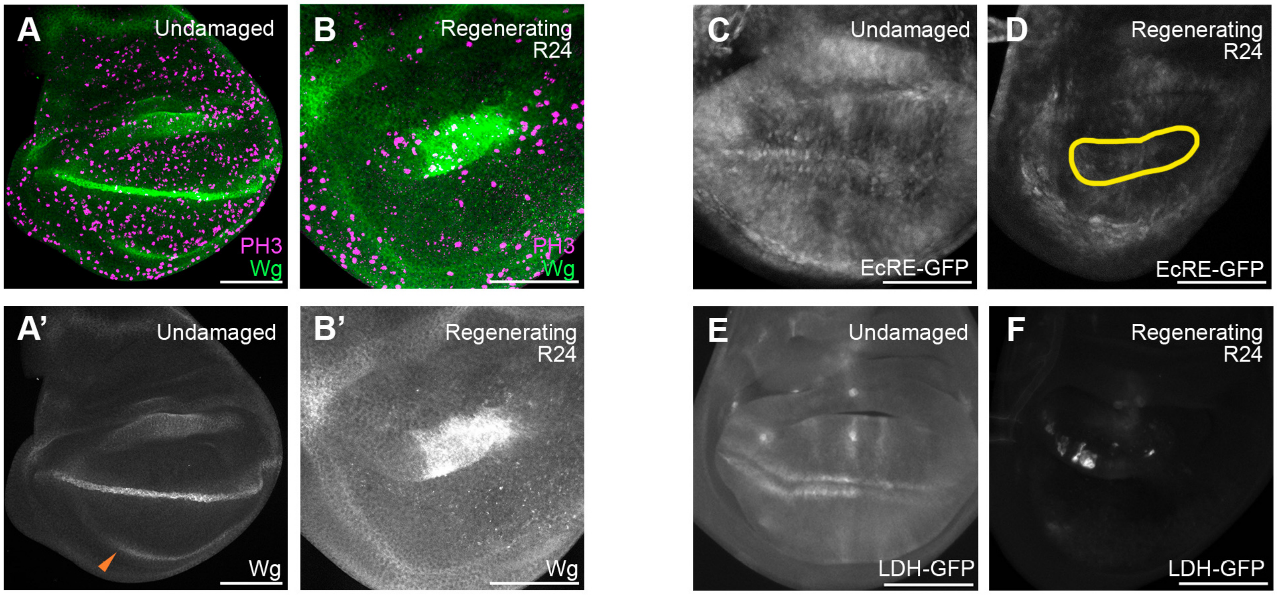

Fig. S1. Ecdysone receptor and Lactate dehydrogenase do not drive regenerative growth

(A-B') PH3 and Wg staining in an undamaged disc (A-A') and an R24 disc (B-B'). Orange arrowhead in (A') indicates inner ring of Wg. Wg staining was used to draw the yellow line in Fig. 1F and identify the pouch area in Fig. 1E. (C-D) EcRE-GFP expression in an undamaged disc (C) and an R24 disc (D). (E-F) LDH-GFP expression in an undamaged disc (E) and an R24 disc (F).

Development • Supplementary information

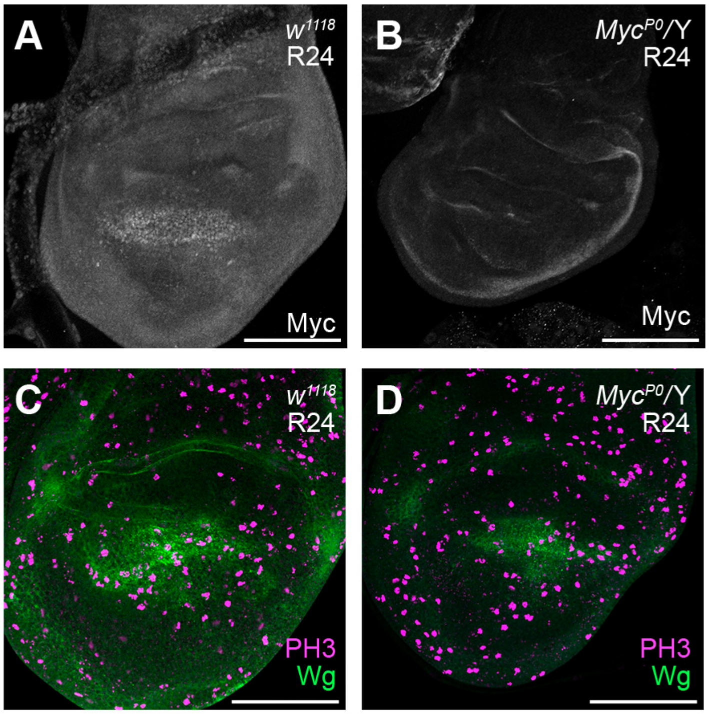

Fig. S2. The *Myc^{P0}* mutation reduces Myc and proliferation during regeneration (A-B) Myc immunostaining in a *w^{1118}* R24 disc (A) and a *Myc^{P0}/Y* R24 disc (B). (C-D) PH3 and Wg staining in a *w^{1118}* R24 disc (C) and a *Myc^{P0}/Y* R24 disc (D). The Wg staining was used to draw the yellow lines in Fig. 2D,E.

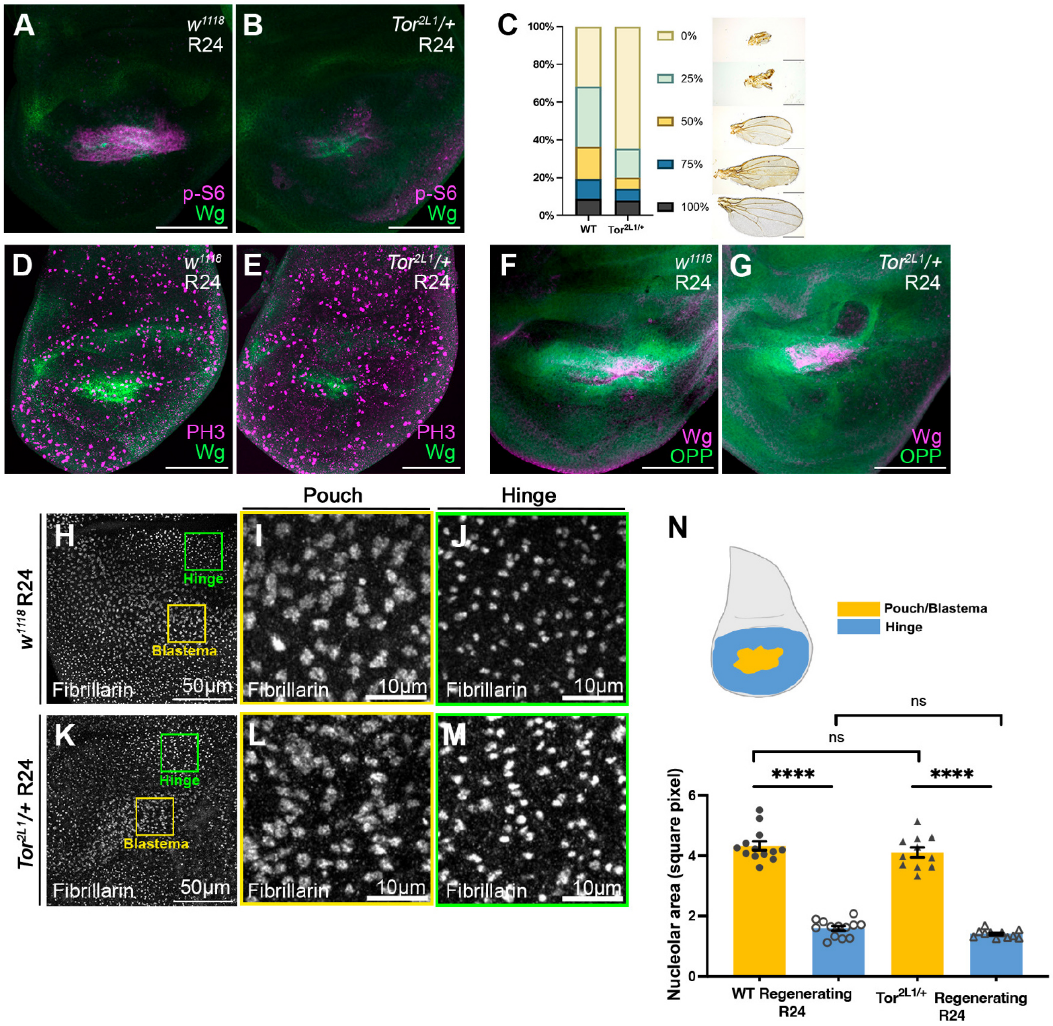

Fig. S3. Effects of the *Tor*^{2L1} mutation on regeneration.

(A-B) p-S6 and Wg staining in a *w*^{1118} R24 disc (A) and a *Tor*^{2L1}/+ R24 disc (B). The Wg staining was used to draw the yellow lines in Fig. 3A,B. (C) Size of adult wings after disc regeneration in *w*^{1118} and *Tor*^{2L1}/+ animals. Scale bars are 500µm. (D-E) PH3 and Wg staining in a *w*^{1118} R24 disc (D) and a *Tor*^{2L1}/+ R24 disc (E). The Wg staining was used to draw the yellow lines in Fig. 3G,H. (F-G) OPP assay with Wg staining in a *w*^{1118} R24 disc (F) and a *Tor*^{2L1}/+ R24 disc (G). The Wg staining was used to draw the yellow lines in Fig. 3J,K. (H-M) Fibrillarin staining marking nucleoli in a *w*^{1118} R24 disc (H-J) and a *Tor*^{2L1}/+ R24 disc (K-M). (I, L) 200-pixel^2 area from blastema in H, K accordingly (yellow boxes), identified by Wg staining. (J, M) 200-pixel^2 area from wing hinge in H, K (green boxes). (N) Quantification of nucleolus size in hinge and blastema. *w*^{1118} n=13, *Tor*^{2L1}/+ n=11. ****P<0.0001, n.s. P>0.05. Scale bars are 100µm unless otherwise marked. Error bars are SEM. Statistical test used was Welch's t-test.

Development • Supplementary information

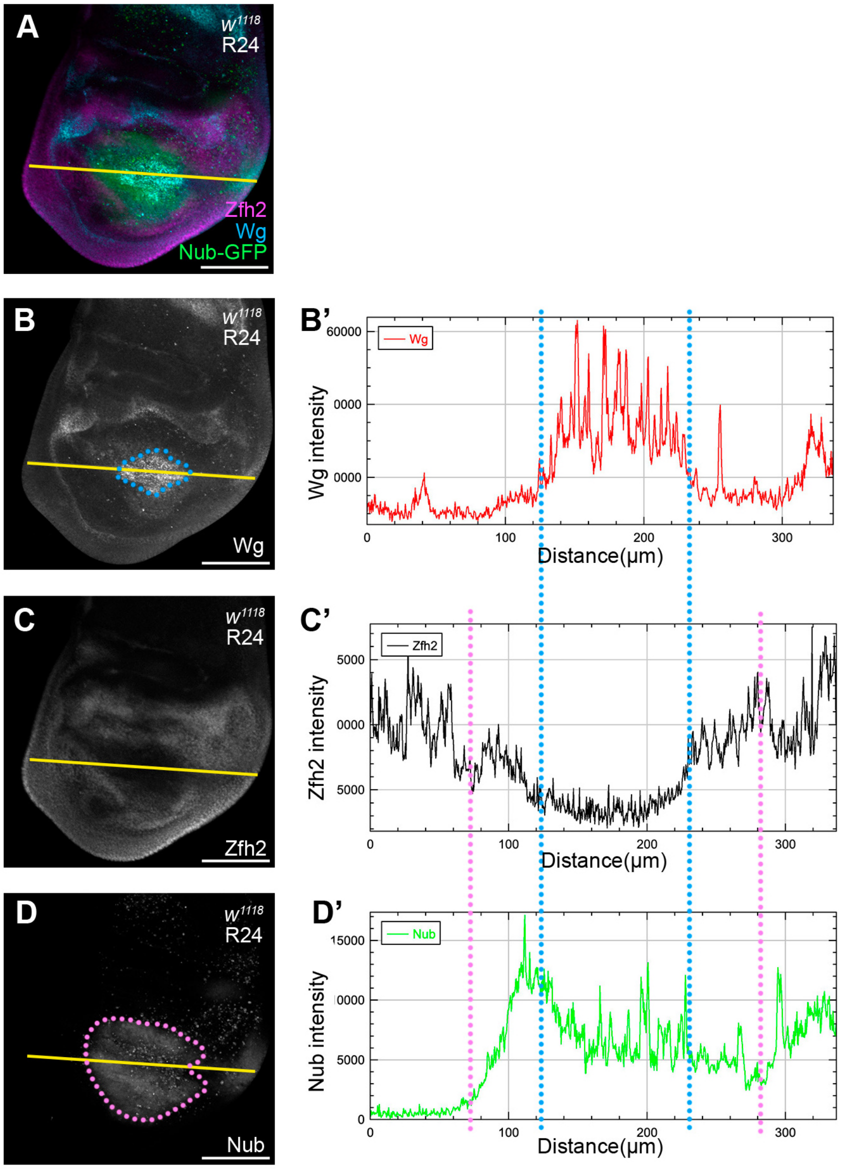

Fig. S4. Map of Zfh2, Wg, and Nub expression.

(A) R24 disc expressing *nub-GFP* with Zfh2 and Wg immunostaining as in Fig. 4L. (B) Wg staining. (B') Intensity plot of Wg staining taken from the yellow cross-section line in (B). (C) Zfh2 staining. (C') Intensity plot of Zfh2 staining taken from the yellow cross-section line in (C). (D) *nub-GFP* expression. (D') Intensity plot of GFP taken from the yellow cross-section line in (D). Blue dotted circle in (B) and blue dotted lines in (B'-D') indicate the edge of Wg-expressing area. Pink dotted circle in (D) and pink dotted lines in (C'-D') indicate edge of *nub-GFP* expressing area. Scale bars are 100μm.

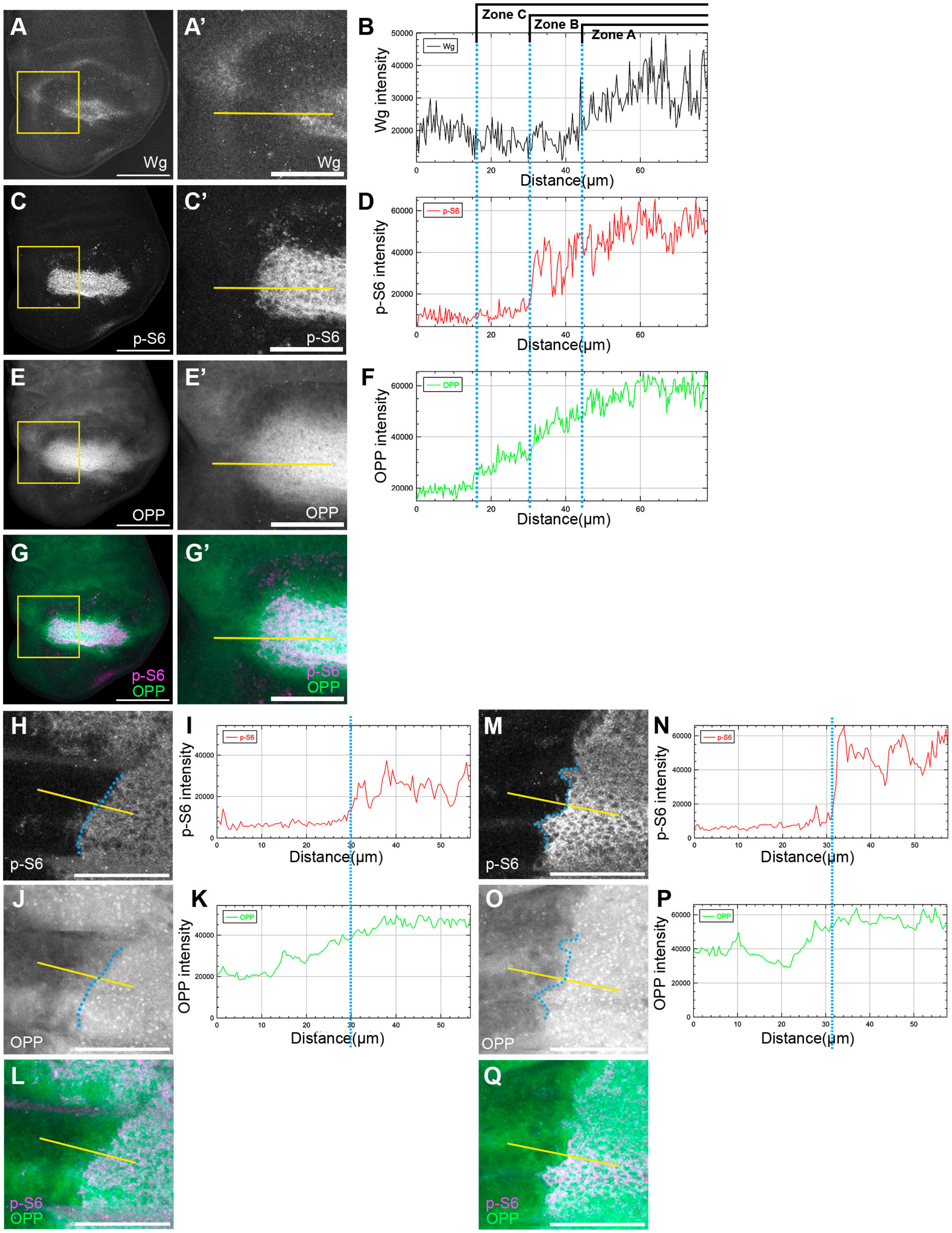

A
A'
Wg
B
Zone C
Zone B
Zone A
Wg intensity
Distance(μm)
C
C'
p-S6
D
p-S6 intensity
E
E'
OPP
F
OPP intensity
G
G'
H
I
J
K
L
M
N
O
P
Q

Fig. S5. Map of Wg, p-S6, and OPP in the blastema.

(A,C,E,G) A *w[1118]* R24 disc with Wg (A) and p-S6 (C) staining, OPP assay (E), and merge of p-S6 and OPP (G). (A’,C’,E’,G’) A higher magnification image of the yellow square in (A-G). (B,D,F) Fluorescence intensity plots taken from the yellow line in (A’,C’,E’). (H-Q) *hhGal4, UAS-Tsc2RNAi* (*hh>Tsc2i*) undamaged discs with p-S6 immunostaining (H, M) and OPP assay (J, O). Note (H-K) are from one wing disc and (M-Q) are from another wing disc. (I) Fluorescence intensity plot taken from the yellow line in (H). (K) Fluorescence intensity plot taken from the yellow line in (J). (N) Fluorescence intensity plot taken from the yellow line in (M). (O) Fluorescence intensity plot taken from the yellow line in (P). Blue dotted lines in (H-K) and (M-P) indicate the edge between high/low p-S6 staining. Scale bars are 100μm.

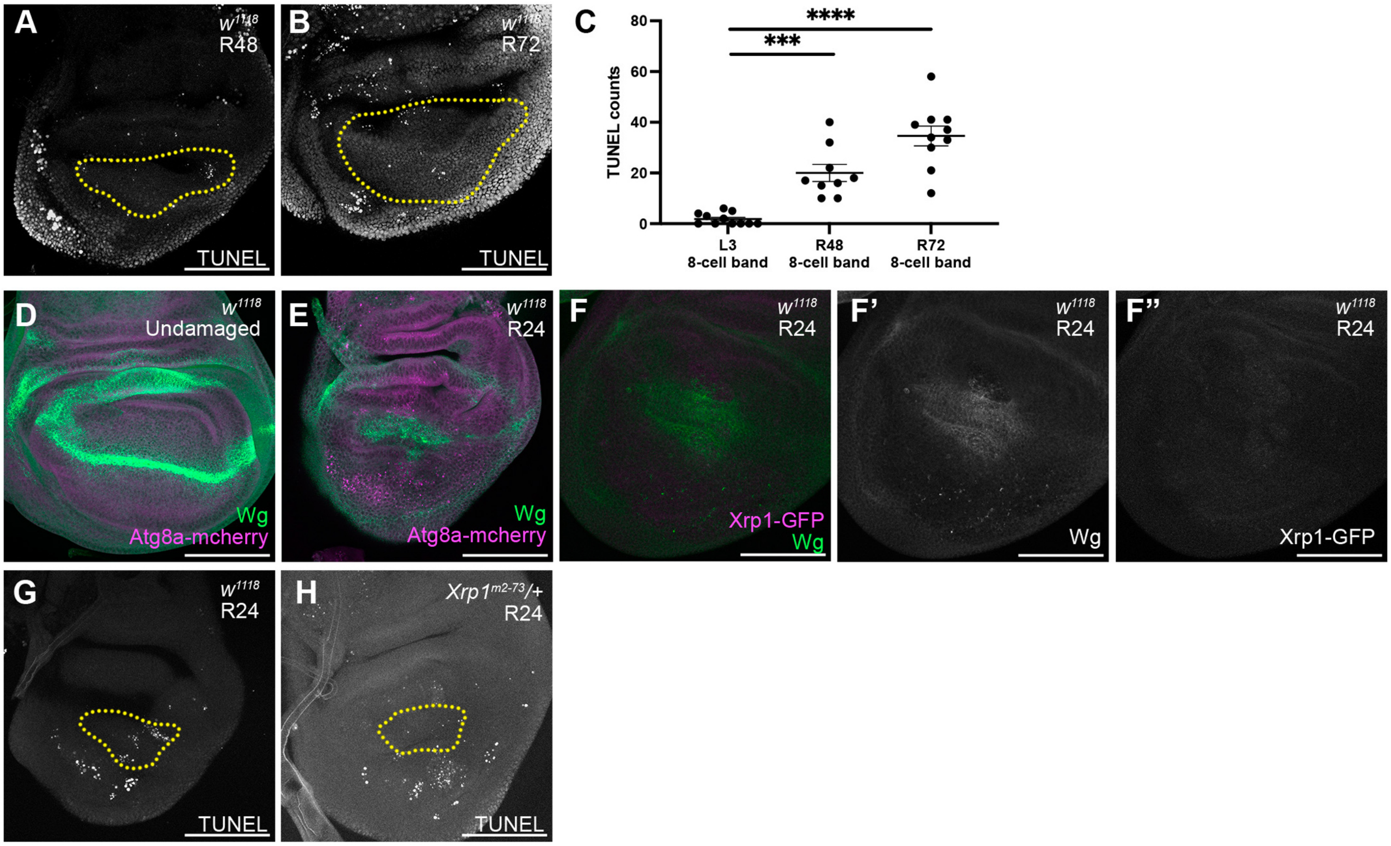

Fig. S6. Cell death at later time points and expression of Atg8a and Xrp1-GFP. (A-B) TUNEL assay marking apoptotic cells in an R48 wing disc (A) and an R72 wing disc (B). Yellow dotted circle indicates Zone A marked by Myc. (C) Quantification of cell death (TUNEL) in the 8-cell zone adjacent to the Myc+ area. L3 n=11, R48 n=9, R72 n=10. ***P<0.001, ****P<0.0001. Error bars are SEM. Statistical test used was Welch's t-test. (D-E) Atg8a-mcherry expression in an undamaged disc (D) and an R24 disc (E). (F-F") Xrp1-GFP expression in wing disc with Wg staining. (F) merge, (F') Wg, (F'') GFP. (G-H) TUNEL assay marking apoptotic cells in a w^{1118} R24 disc (G) and a *Xrp1*$^{M2-73}$/+ disc (H). Yellow dotted circle indicates Growth Zone A marked by Wg. Scale bars are 100µm.

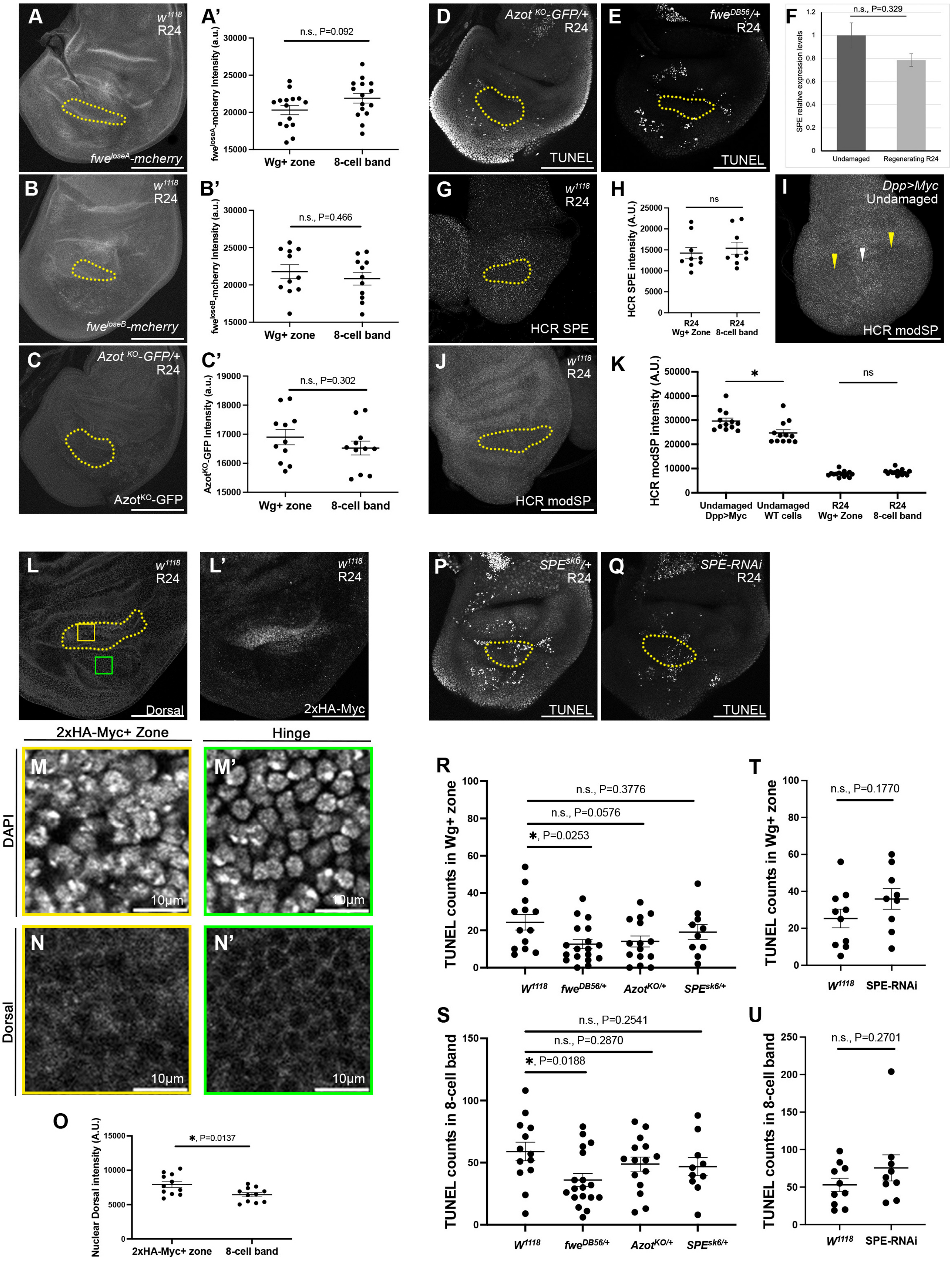

A
w1118 R24
fweloseA-mcherry
A'
n.s., P=0.092
Wg+ zone
8-cell band
B
B'
n.s., P=0.466
fweloseB-mcherry
C
Azot KO-GFP/+ R24
AzotKO-GFP
C'
n.s., P=0.302
D
E
fweDB56/+ R24
TUNEL
F
n.s., P=0.329
SPE relative expression levels
Undamaged
Regenerating R24
G
HCR SPE
H
ns
HCR SPE intensity (A.U.)
I
Dpp>Myc Undamaged
HCR modSP
J
K
HCR modSP intensity (A.U.)
Undamaged Dpp>Myc
Undamaged WT cells
R24 Wg+ Zone
R24 8-cell band
L
L'
Dorsal
2xHA-Myc
2xHA-Myc+ Zone
Hinge
DAPI
M
M'
N
N'
10µm
O
*, P=0.0137
Nuclear Dorsal intensity (A.U.)
2xHA-Myc+ zone
8-cell band
P
SPEsk6/+ R24
Q
SPE-RNAi R24
R
n.s., P=0.3776
n.s., P=0.0576
*, P=0.0253
TUNEL counts in Wg+ zone
W1118
fweDB56/+
AzotKO/+
SPEsk6/+
T
n.s., P=0.1770
SPE-RNAi
S
n.s., P=0.2541
n.s., P=0.2870
*, P=0.0188
TUNEL counts in 8-cell band
U
n.s., P=0.2701

Fig. S7. Azot expression and Toll signaling do not regulate cell competition during regeneration.

(A) *fwe*^{loseA}-mcherry expression in an R24 disc. (A') Quantification of average mCherry intensity. n=15. (B) *fwe*^{loseB}-mcherry expression in an R24 disc. (B') Quantification of average mCherry intensity. n=11. (C) *azot*^{KO}*-GFP* expression in an R24 disc. (C') Quantification of average GFP intensity. n=11. (D-E) TUNEL assay marking apoptotic cells in an *azot*$^{KO\text{-}GFP}$/+ disc (D) and an *fwe*^{DB56}/+ disc (E). (F) RT-qPCR of SPE in undamaged and R24 wing discs. (G) HCR of *SPE* in an R24 disc. (H) Quantification of *SPE* intensity n=9. n.s. p>0.05. (I-J) HCR of *modSP* in an undamaged Dpp>Myc wing disc (I) and an R24 wing disc (J). (I) White arrowhead marks the high-Myc cells and yellow arrowheads mark the wildtype cells. (K) Quantification of *modSP* intensity. Dpp>Myc n=12. R24 *w*^{1118} n=14. n.s. p>0.05, *p<0.05. (L-N') A R24 wing disc with Dorsal staining (L) and with 2xHA-Myc (L'). Yellow dotted circle indicates Growth Zone A marked by 2xHA-Myc+ cells. (M, N) 100-pixel^2 area from Zone A in (L) (yellow box) with DAPI staining (M) and Dorsal staining (N). (M', N') 100-pixel^2 area from the 8-cell zone in (L) (green box) with DAPI staining (M') and Dorsal staining (N'). (O) Quantification of Dorsal intensity in Growth Zone A and in the 8-cell zone adjacent to Growth Zone A. n=11. *p<0.05. (P-Q) TUNEL assay marking apoptotic cells in an *SPE*^{sk6}/+ disc (P) and an *SPE-RNAi* disc (Q). (R) Quantification of cell death in the Wg + Zone A. (S) Quantification of cell death in the eight-cell band outside Zone A in R24 discs. *w*^{1118} n=13, *fwe*^{DB56}/+ n=18, *azot* ^{KO}/+ n=15, *SPE*^{sk6}/+ n=10. (T) Quantification of cell death in the Wg+ Zone A. *w*^{1118} n=10, *SPE-RNAi* n=9. (U) Quantification of cell death in the eight-cell band outside Zone A in R24 discs. *w*^{1118} n=10, *SPE-RNAi* n=9. Yellow dotted circle in A-E, G, J, P-Q indicates growth zone A marked by Wg. Scale bars are 100µm unless otherwise marked. Error bars are SEM. Statistical tests were Welch's t-test.

Development • Supplementary information
